# Catalogue of NIMS fatigue data sheets

**DOI:** 10.1080/14686996.2019.1680574

**Published:** 2019-10-21

**Authors:** Yoshiyuki Furuya, Hideaki Nishikawa, Hisashi Hirukawa, Nobuo Nagashima, Etsuo Takeuchi

**Affiliations:** Research Center for Structural Materials, National Institute for Materials Science, Tsukuba, Japan

**Keywords:** Fatigue, structural materials, database, room temperature, high temperature, welded joint, 106 Metallic materials, Mechanical properties

## Abstract

This paper summarizes the NIMS fatigue data sheets and makes a new gateway available to access them. The NIMS fatigue data sheets are a huge database of the fatigue properties of structural materials. This project covers fundamental fatigue properties at room temperature and at high temperatures, and the fatigue properties of welded joints. The fundamental fatigue properties recorded include high-cycle, low-cycle and gigacycle fatigue test results for steels, aluminium alloys, titanium alloys and so on. The high-cycle fatigue test results determine the fatigue limits. The low-cycle fatigue test results reveal not only the fatigue lives but also cyclic stress-strain curves. The gigacycle fatigue tests were conducted at 100 Hz for three years up to 10^10^ cycles, as well as at 20 kHz for a week. The fatigue properties at high temperatures were evaluated chiefly for steels, via low and high-cycle fatigue tests. The low-cycle fatigue tests were conducted by employing various strain rates and waveforms. The fatigue properties of welded joints were evaluated using thick plates of steels and aluminium alloys, conducting high-cycle fatigue and crack propagation tests employing large specimens in as-welded condition. The high-cycle fatigue tests were conducted using various specimen sizes, welding procedures, stress ratios and so on. The crack propagation tests were conducted for the base metal, the weld metal and the heat-affected zone. Many new findings were obtained with these fatigue data as reviewed in this paper.

## GRAPHICAL ABSTRACT

**Figure:**
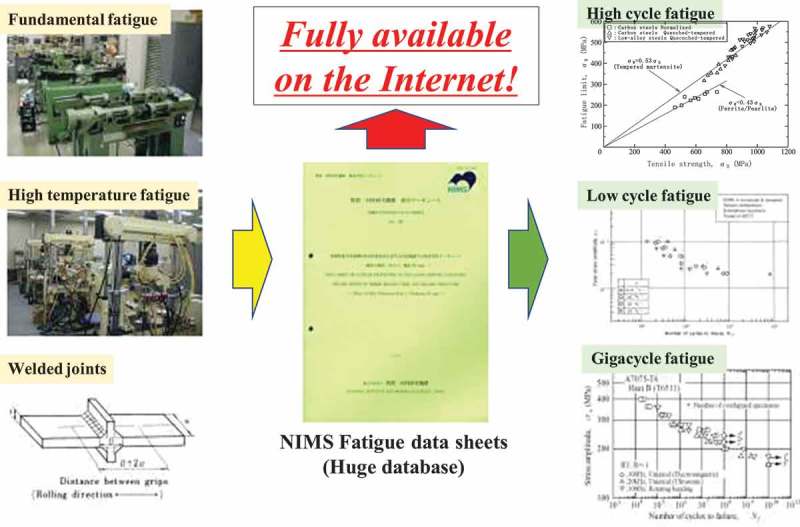

## 1. Introduction

NIMS fatigue data sheets comprise a huge database of fatigue properties of structural materials. The total number of fatigue data sheets is 126 to date and is still increasing. They are distributed free of charge as printed documents. Previously, the content could be viewed on the NIMS website, but downloading and printing were prohibited. As of the publication of this review paper, however, the NIMS fatigue data sheets are fully available on the Internet. The fatigue data sheets are temporally only viewed on ‘MatNavi’ (https://smds.nims.go.jp/fatigue/index_en.html) until the middle of 2020, while they will be fully available on a ‘Materials Data Repository (MDR)’ system managed by NIMS after that.

The first issue of fatigue data sheets was published in 1978, so this undertaking has a history of over 40 years. The following three subjects were initially selected for the project.

1. The fundamental fatigue properties of metallic materials at room temperature in air.
2. The fatigue properties of heat-resistant metallic materials at high temperatures.
3. The fatigue properties of welded joints.

This project was briefly interrupted in 1995, but it was restarted in 1996. After the restart, long-term fatigue was a common theme in each subject. When examining fundamental fatigue properties, for example, the cut-off cycles of the fatigue tests were extended from 10^7^ to 10^10^. Although the high-temperature fatigue tests were conducted under strain-controlled conditions in low-cycle regions, the strain-controlled tests were continued up to high-cycle regions of 10^6^ cycles. The fatigue tests for the welded joints were conducted mainly at 10 Hz, so the cut-off cycles were normally 10^6^: these were extended to 10^8^. As a result, the fatigue tests took several months or years.

The materials tested were made in Japan. Most were JIS-grade materials. Specific types of materials were systematically selected to cover the major grades of the materials. They were directly sampled from the manufacturer.

All the fatigue tests were conducted by NIMS and its predecessor (NRIM: National Research Institute for Metals). This means that the fatigue test conditions were standardized down to the smallest detail. For example, the same types of testing machines were used, the specimens were machined in the same manner, and all the tests were performed by experienced technicians. As the result, data scattering is very low in the NIMS fatigue data sheets. This is one of the major advantages of this database.

The NIMS fatigue data sheets disclose fatigue test results as numerical data. Numerical data remain fully relevant even if how they are interpreted develops in the light of scientific and technological progress. The fatigue test results were validated by an external technical advisory committee whose members consist of industrial engineers currently active in their field. The technical advisory committee also supports the planning of fatigue tests, and the final form of the fatigue data sheets was validated by an internal editorial committee.

The NIMS fatigue data sheets have thus been produced over a long period and maintain a high level of quality. The contents are reviewed below.

## 2. Content

The published NIMS fatigue data sheets are listed in Tables 1–3. No. 0 of the fatigue data sheets [1] is an explanation of the project and No. 84 [36] is a catalogue of the fatigue data sheets of Nos. 1–83. The general contents are summarized as follows.

**Table 1.** List of data sheets for fundamental fatigue properties at room temperature.

No.	Title	Reference
1	Fatigue properties of S25C (0.25C) steel for machine structural use	[2]
2	Fatigue properties of S35C (0.35C) steel for machine structural use	[3]
3	Fatigue properties of S45C (0.45C) steel for machine structural use	[4]
4	Fatigue properties of S55C (0.55C) steel for machine structural use	[5]
8	Fatigue properties of SCr440 (0.40C-1Cr) steel for machine structural use	[6]
9	Fatigue properties of SCM435 (0.35C-1Cr-0.2Mo) steel for machine structural use	[7]
10	Fatigue properties of SCM440 (0.20C-1Cr-0.2Mo) steel for machine structural use	[8]
16	Fatigue properties of SMn438 (0.38C-1.5Mn) steel for machine structural use	[9]
17	Fatigue properties of SMn443 (0.43C-1.5Mn) steel for machine structural use	[10]
24	Fatigue properties of SNC631 (0.31C-2.7Ni-0.8Cr) steel for machine structural use	[11]
25	Fatigue properties of SNCM439 (0.39C-1.8Ni-0.8Cr-0.2Mo) steel for machine structural use	[12]
26	Fatigue properties of SNCM447 (0.47C-1.8Ni-0.8Cr-0.2Mo) steel for machine structural use	[13]
29	Fatigue properties of SUS430 (17Cr) stainless steel bars for machine structural use	[14]
30	Fatigue properties of SUS403 (12Cr) stainless steel bars for machine structural use	[15]
33	Fatigue properties of SUS304 (18Cr-8Ni) stainless steel bars for machine structural use	[16]
37	Fatigue properties of SCr420 (0.20C-1Cr) carburizing steel for machine structural use	[17]
38	Low-cycle fatigue properties of S25C (0.25C) steel for machine structural use	[18]
39	Low-cycle fatigue properties of S35C (0.35C) steel for machine structural use	[19]
43	Fatigue properties of SCM420 (0.20C-1Cr-0.2Mo) carburizing steel for machine structural use	[20]
44	Low-cycle fatigue properties of S45C (0.45C) steel for machine structural use	[21]
45	Low-cycle fatigue properties of SCr440 (0.40C-1Cr) steel for machine structural use	[22]
50	Fatigue properties of SNCM220 (0.20C-0.5Ni-0.5Cr-0.2Mo) carburizing steel for machine structural use	[23]
51	Fatigue properties of SNCM420 (0.20C-1.8Ni-0.5Cr-0.2Mo) carburizing steel for machine structural use	[24]
52	Low-cycle fatigue properties of SCM435 (0.35C-1Cr-0.2Mo) steel for machine structural use	[25]
56	Low-cycle fatigue properties of SNCM439 (0.39C-1.8Ni-0.8Cr-0.2Mo) steel for machine structural use	[26]
59	High-cycle fatigue properties of SUP7 (2.0Si-0.8Mn) steel for springs	[27]
60	High-cycle fatigue properties of SUP9A (0.8Mn-0.8Cr) steel for springs	[28]
61	Low-cycle fatigue properties of A5083P-O (Al-4.5Mg-0.6Mn) aluminium alloy plates	[29]
63	High-cycle fatigue properties of SUP12 (1.4Si-0.7Cr) steel for springs	[30]
69	High-cycle fatigue properties of SKD61 (0.36C-5Cr-1.25Mo-1V) steel for tools	[31]
70	Low-cycle fatigue properties of A7N01S-T5 (Al-4.6Zn-1.2Mg) aluminium alloy extruded shapes	[32]
73	High-cycle fatigue properties off SKD11 (1.5C-12Cr-1Mo-0.35V) steel for tools	[33]
74	Low-cycle fatigue properties of A7N01P-T6 (Al-4.5Zn-1.5Mg) aluminium alloy plates	[34]
83	Data sheets on elastic moduli of steels	[35]
85	Fatigue properties of Ti-6Al-4V (900 MPa class) titanium alloy	[37]
87	Giga-cycle fatigue properties of SUP7 (2.0Si-0.8Mn) steel for springs	[38]
89	Fatigue properties of Ti-6Al-4V (1100 MPa class) titanium alloy	[39]
92	Giga-cycle fatigue properties of Ti-6Al-4V (900 MPa class) titanium alloy	[40]
93	Ultrasonic fatigue properties of SUP7 (2.0Si-0.8Mn) steel for springs	[41]
95	Fatigue properties of pure titanium	[42]
97	Giga-cycle fatigue properties of S40C (0.40C) carbon steel for machine structural use	[43]
98	Giga-cycle fatigue properties of Ti-6Al-4V (1100 MPa class) titanium alloy	[44]
101	Fatigue properties of Ti-6Al-4V ELI (900 MPa class) titanium alloy	[45]
102	Ultrasonic fatigue properties of S40C (0.40C) carbon steel for machine structural use	[46]
103	Fatigue properties of Ti-6Al-4V ELI (1100 MPa class) titanium alloy	[47]
104	Giga-cycle fatigue properties of SCM440 (0.40C-1Cr-0.2Mo) steel for machine structural use	[48]
105	Giga-cycle fatigue properties of Ti-6Al-4V ELI (900 MPa class) titanium alloy	[49]
106	Ultrasonic fatigue properties of SCM440 (0.40C-1Cr-0.2Mo) steel for machine structural use	[50]
107	Giga-cycle fatigue properties of Ti-6Al-4V ELI (1100 MPa class) titanium alloy	[51]
110	Fatigue properties of extruded AZ61(Mg-6Al-1Zn) and AZ31(Mg-3Al-1Zn) magnesium alloys	[52]
111	Giga-cycle fatigue properties of Ti-6Al-4V (900 MPa class) titanium alloy at high stress ratios	[53]
112	Giga-cycle fatigue properties of SUJ2 (1.0C-1.5Cr) steel for bearings	[54]
115	Giga-cycle fatigue properties of Ti-6Al-4V ELI (900 MPa class) titanium alloy at high stress ratios	[55]
116	Giga-cycle fatigue properties of hydrogen charged SCM440 (0.4C-1Cr-2Mo) steel for machine structural use	[56]
117	Giga-cycle fatigue properties of FCD400 and FCD800 spheroidal graphite cast iron	[57]
118	Low- and high-cycle fatigue properties of SUS630 (16Cr-4Ni-4Cu) stainless steel	[58]
119	Giga-cycle fatigue properties of A5083P-O (Al-4.5Mg-0.6Mn) aluminium alloy plates	[59]
120	Giga-cycle fatigue properties of SUS630 (16Cr-4Ni-4Cu) stainless steel	[60]
121	Giga-cycle fatigue properties of A5083P-O (Al-4.5Mg-0.6Mn) aluminium alloy plates at high stress ratios	[61]
122	Giga-cycle fatigue properties of SUS630 (16Cr-4Ni-4Cu) stainless steel at high stress ratios	[62]
123	Low-and high-cycle fatigue properties of A7075-T6 (Al-5.6Zn-2.5Mg-1.6Cu) aluminium alloy	[63]
124	Low- and high-cycle fatigue properties of SUS329J3L (22Cr-5Ni-3Mo) duplex stainless steel	[64]
125	Giga-cycle fatigue properties of A7075-T6 (Al-5.6Zn-2.5Mg-1.6Cu) aluminium alloy	[65]

**Table 2.** List of data sheets for fatigue properties at high temperature.

No.	Title	Reference
6	Elevated-temperature, high-cycle fatigue properties of SUS403-B (12Cr) stainless steel bar for turbine blades	[66]
7	Elevated-temperature, low-cycle fatigue properties of SCMV4 (2.25Cr-1Mo) steel plate for pressure vessels	[67]
14	Elevated-temperature, high-cycle fatigue properties of S45C (0.45C) steel for machine structural use	[68]
15	Elevated-temperature, high-cycle and low-cycle fatigue properties of SUS316-HP (18Cr-12Ni-2Mo) hot rolled stainless steel plate	[69]
22	Elevated-temperature, low-cycle fatigue properties of SB49 carbon steel plate for boilers and other pressure vessels	[70]
23	Elevated-temperature, high-cycle fatigue properties of SCM435 (0.35C-1Cr-0.2Mo) steel for machine structural use	[71]
28	Elevated-temperature, low-cycle fatigue properties of SCMV3 (1.25Cr-0.5Mo) steel plate for pressure vessels	[72]
32	Elevated-temperature, high-cycle fatigue properties of NCF800H (Fe-21Cr-32Ni-Ti-Al) alloy bar for corrosion and heat resisting applications	[73]
35	Elevated-temperature, high-cycle fatigue properties of SUH616-B (12Cr-1Mo-1W-0.3V) heat-resisting steel bar	[74]
36	Elevated-temperature, time-dependent low-cycle fatigue properties of NCF800H-B (Fe-21Cr-32Ni-Ti-Al) alloy bar for corrosion and heat-resisting applications	[75]
42	Elevated-temperature, high-cycle fatigue properties of SUS304-HP (18Cr-8Ni) hot rolled stainless steel plate	[76]
48	Elevated-temperature, high-cycle fatigue properties of SCMV4 (2.25Cr-1Mo) steel plate for pressure vessels	[77]
49	Elevated-temperature, time-dependent low-cycle fatigue properties of SUS304-HP (18Cr-8Ni) hot rolled stainless steel plate	[78]
55	Elevated-temperature, high-cycle fatigue properties of ASTM A470-8 (1Cr-1Mo-0.25V) steel forging for turbine rotors and shafts	[79]
58	Elevated-temperature, time-dependent low-cycle fatigue properties of ASTM A470-8 (1Cr-1Mo-0.25V) steel forging for turbine rotors and shafts	[80]
62	Elevated-temperature, time-dependent low-cycle fatigue properties of SCMV4 (2.25Cr-1Mo) steel plate for pressure vessels	[81]
66	High-cycle fatigue properties of SB46 carbon steel plate for boilers and other pressure vessels at intermediate temperatures	[82]
67	Low-cycle fatigue properties at elevated temperatures for weld and base metals of SB450 carbon steel plate for boilers and other pressure vessels	[83]
68	Elevated-temperature, time-dependent low-cycle fatigue properties of SUH616-B (12Cr-1Mo-1W-0.3V) heat-resisting steel bar	[84]
72	Elevated-temperature, high-cycle fatigue properties of SCMV2-2 NT (1Cr-0.5Mo) low alloy steel plate for boilers and other pressure vessels	[85]
75	Elevated-temperature fatigue properties for butt welded joints of SCMV2-2 NT (1Cr-0.5Mo) low alloy steel plate for boilers and other pressure vessels	[86]
77	Low-cycle fatigue properties at elevated temperatures for weld and base metals of SCMV2-2 NT (1Cr-0.5Mo) low alloy steel plate for boilers and other pressure vessels	[87]
78	Elevated-temperature, time-dependent low-cycle fatigue properties of ASTM A387 grade 91 (9Cr-1Mo) steel plate for pressure vessels	[88]
79	Elevated-temperature fatigue properties for butt welded joints of SB450 carbon steel plate for boilers and other pressure vessels	[89]
81	Elevated temperature fatigue crack propagation properties for butt welded joints of SCMV2-2 NT (1Cr-0.5Mo) low alloy steel plate for boilers and other pressure vessels	[90]
82	Elevated temperature fatigue crack propagation properties for butt welded joints of SB450 carbon steel plate for boilers and other pressure vessels	[91]
86	Long-term, high temperature low-cycle fatigue properties of ferritic heat-resisting steel plate (12Cr-2W)	[92]
88	Long-term, high temperature low-cycle fatigue properties of ferritic heat-resisting steel plate (9Cr-2W)	[93]
94	Long-term, high temperature low-cycle fatigue properties of SCMV4 (2.25Cr-1Mo) steel plate for boilers and pressure vessels	[94]
100	Long-term, high temperature low-cycle fatigue properties of SUS310S (25Cr-20Ni) hot rolled stainless steel plate	[95]
109	Long-term, high temperature low-cycle fatigue properties of NCF800H (21Cr-32Ni-Ti-Al) corrosion-resisting and heat-resisting alloy plate	[96]
113	Long-term, high temperature low-cycle fatigue properties of NW6617 (Ni-22Cr-12Co-9Mo) nickel alloy plate	[97]

**Table 3.** List of data sheets for fatigue properties of welded joints.

No.	Title	Reference
5	Fatigue properties for butt-welded joints of SM50B high tensile structural steel plates	[98]
11	Fatigue properties of butt-welded joints of SM58Q rolled steel for welded structure – effect of specimen size -	[99]
12	Fatigue properties of butt-welded joints of high strength steel (class 800 N/mm^2^) for welded structure – effect of specimen size -	[100]
13	Fatigue properties of non-load-carrying cruciform welded joints of SM50B rolled steel for welded structure – effect of specimen size -	[101]
18	Fatigue properties for load-carrying cruciform welded joints of SM50B rolled steel for welded structure – effect of specimen size -	[102]
19	Fatigue properties for butt welded joints of high strength steel (class 800 N/mm^2^) for welded structure – effect of welding procedure -	[103]
20	Fatigue properties for non-load-carrying cruciform welded joints of SM50B rolled steel for welded structure – effect of welding procedure -	[104]
21	Fatigue crack propagation properties for butt welded joints of SM50B rolled steel for welded structure – effect of welding procedure -	[105]
27	Fatigue properties for butt welded joints of SM50B rolled steel for welded structure – effect of welding procedure -	[106]
31	Fatigue crack propagation properties for butt welded joints of high strength steel (class 800 N/mm^2^) for welded structure – effect of welding procedure -	[107]
34	Fatigue properties for butt welded joints of SB42 carbon steel plate for boilers and other pressure vessels – effect of stress ratio -	[108]
40	Fatigue properties for butt welded joints of SPV50 steel plate for pressure vessels – effect of stress ratio -	[109]
41	Fatigue crack propagation properties for butt welded joints of SB42 carbon steel plate for boilers and other pressure vessels – effect of stress ration -	[110]
46	Fatigue crack propagation properties for butt welded joints of SPV50 (Si-Mn, 500 N/mm^2^ YS) steel plate for pressure vessels – effect of stress ration -	[111]
47	Fatigue properties for weld and HAZ materials of SPV50 (Si-Mn, 500 N/mm^2^ YS) steel plate for pressure vessels	[112]
53	Fatigue properties for butt welded joints of SUS304-HP (18Cr-8Ni) hot rolled stainless steel plate – effect of stress ratio -	[113]
54	Fatigue crack propagation properties for butt welded joints of SUS304-HP (18Cr-8Ni) hot rolled stainless steel plate – effect of stress ratio -	[114]
57	Fatigue properties for weld and HAZ materials of SB42 (C-Si, 420 N/mm^2^ TS) carbon steel plate for boilers and other pressure vessels	[115]
64	Fatigue properties for butt welded joints of A5083P-O (Al-4.5Mg-0.6Mn) aluminium alloy plates	[116]
65	Fatigue properties for weld and base metals of SUS304-HP (18Cr-8Ni) hot rolled stainless steel plate	[117]
71	Fatigue properties of butt welded joints of A7N01S-T5 (Al-4.6Zn-1.2Mg) aluminium alloy extruded shapes	[118]
76	Fatigue properties for butt welded joints of A7N01P-T6 (Al-4.5Zn-1.5Mg) aluminium alloy plates	[119]
80	Fatigue properties for butt welded joints of A6N01S-T5 (Al-0.6Mg-0.65Si) aluminium alloy extruded shapes	[120]
90	Fatigue properties of non-load-carrying cruciform welded joints of SM570Q rolled steel for welded structure – effect of residual stress -	[121]
91	Fatigue properties of non-load-carrying cruciform welded joints of SM490B rolled steel for welded structure – effect of residual stress -	[122]
96	Fatigue properties of non-load-carrying cruciform welded joints of SM490B rolled steel for welded structure – effect of plate thickness (Part 1, thickness 9 mm) -	[123]
99	Fatigue properties of non-load-carrying cruciform welded joints of SM490B rolled steel for welded structure －effect of plate thickness (Part 2, thickness 160 mm) －	[124]
108	Fatigue properties of non-load-carrying cruciform welded joints of SM490B rolled steel for welded structure – effect of plate thickness (Part 3, thickness 80 mm) -	[125]
114	Fatigue properties of non-load-carrying cruciform welded joints of SM490B rolled steel for welded structure －effect of plate thickness (Part 4, thickness 40 mm) －	[126]

### 2.1. Fundamental fatigue properties at room temperature in air

The major materials tested were carbon steels, low-alloy steels, stainless steels, high-strength steels, aluminium alloys and titanium alloys. In addition to these materials, magnesium alloys and cast irons are included in the fatigue data sheets Nos. 110 and 117, respectively. To evaluate scattering between heats, more than two heats were sampled and tested for each grade of material. Particularly in the early versions of the fatigue data sheets, more than ten heats were tested for each grade. The processing details of each heat are also disclosed in the fatigue data sheets.

These fatigue data sheets disclose both high- and low-cycle fatigue properties. The high-cycle fatigue tests were conducted under load-controlled conditions. These tests include rotating bending fatigue tests, reversed torsion fatigue tests and uniaxial stress fatigue tests. The low-cycle fatigue tests were conducted under strain-controlled conditions. They consisted not only of constant strain amplitude tests to determine fatigue lives but also incremental step tests [127] to clarify cyclic stress-strain curves.

To study long-term fatigue, fatigue tests up to 10^10^ cycles, so called gigacycle fatigue tests [128], were conducted on high-strength steels, Ti-6Al-4V alloys and aluminium alloys, since fatigue limits were not confirmed in these materials. Two types of testing methods were then employed. One was rotating bending fatigue testing at 100 Hz, which took three years to reach 10^10^ cycles. The other was ultrasonic fatigue testing [129–134] at 20 kHz, which completed the required 10^10^ cycles in a week. Frequency effects can be evaluated by comparing the results between these two types of fatigue tests.

### 2.2. Fatigue properties at high temperatures

The major materials tested were carbon steels, low-alloy steels and stainless steels. A Ni-base alloy is also included in Fatigue Data Sheet No. 113. For high-temperature fatigue tests, a single heat was tested for each grade of the materials, since a greater variety of test conditions is more informative than a greater number of heats. The test conditions consist of temperatures, strain rates, waveforms and so on. The major materials selected were used for pressure vessels.

Although both high- and low-cycle fatigue tests were conducted, the low-cycle fatigue tests under strain-controlled conditions were a major part of the high-temperature fatigue data sheets. The low-cycle fatigue tests consisted of both constant-strain amplitude tests and incremental step tests. Some of the constant-strain amplitude tests include tension hold-type waveforms, i.e., creep-fatigue tests. The high-cycle fatigue tests were conducted as rotating bending fatigue tests in the early versions, and uniaxial stress fatigue tests were added in the later versions. The temperature conditions, which were determined in accordance with the material’s grades, included not only high temperatures of above 400 °C but also intermediate temperatures of below 400 °C.

To investigate long-term fatigue, the strain-controlled fatigue tests were conducted up to high-cycle regions of 10^6^ cycles. In general, the high-cycle fatigue tests were conducted under load-controlled conditions, while the low-cycle fatigue tests were conducted under strain-controlled conditions. The problem was then how to accurately combine these two types of results. The high-cycle fatigue tests under strain-controlled conditions were thus required by industry. However, the strain-controlled tests up to 10^6^ cycles took considerable time: for example, several months were necessary to reach 10^6^ cycles.

### 2.3. Fatigue properties of welded joints

The materials tested were thick plates of carbon steels, low-alloy steels, stainless steels and aluminium alloys. As with the high-temperature fatigue tests, a single heat was tested for each grade of material, since there was a greater demand for a wider range of test conditions. The test conditions included specimen sizes, welding procedures, and stress ratios.

**Figure 1.**
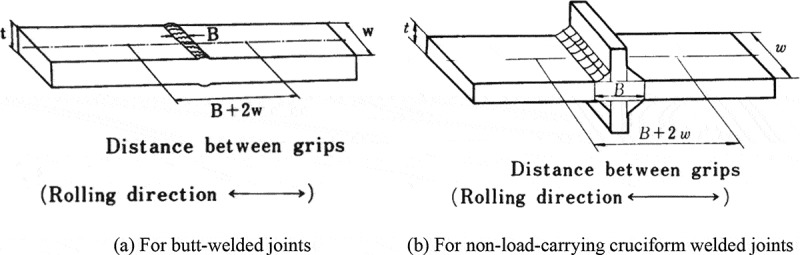
Typical fatigue test specimens used for welded joints.

High-cycle fatigue tests and crack propagation tests were conducted using large specimens. Factors affecting the fatigue properties of welded joints were not only their microstructure and hardness but also shape and residual stress. To include the effects of shape and residual stress, the large specimens contained the welded parts in the as-welded state. Figure 1 shows typical specimens used in the high-cycle fatigue tests. High-capacity fatigue testing machines were needed to test these large specimens.

For long-term fatigue, the high-cycle fatigue tests at 10 Hz were conducted up to 10^8^ cycles, which took several months. The test frequencies for the welded joints ranged from 1–100 Hz and the cut-off cycles were 10^6^ or 10^7^. The fatigue limits were not confirmed by these cut-off cycles, however, so the cut-off cycles were extended. This type of long-term fatigue testing was carried out on non-load-carrying cruciform welded joints of low-alloy steels, to clarify the effects of residual stress and plate thickness.

## 3. Outcome

### 3.1. Fundamental fatigue properties at room temperature in air

**Figure 2.**
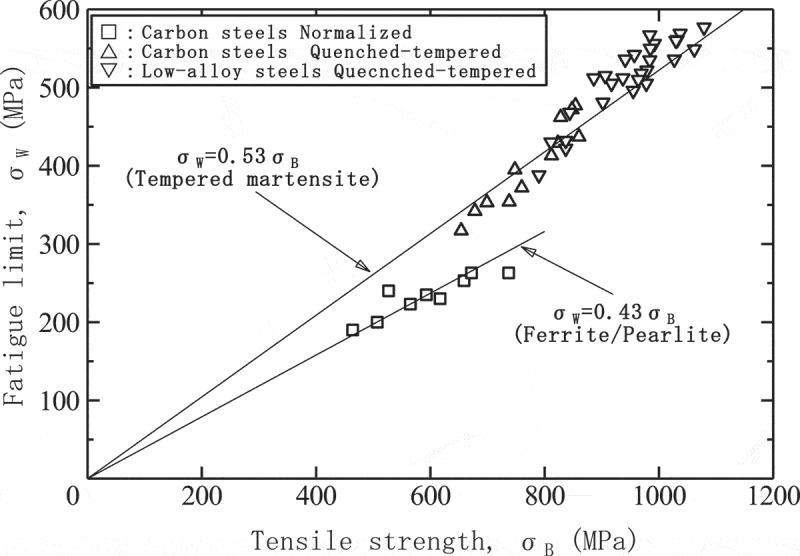
Relationship between fatigue limits and tensile strength for steels.

This series of fatigue data sheets reveals high-cycle fatigue properties, low-cycle fatigue properties and gigacycle fatigue properties. The high-cycle fatigue properties determine the fatigue limits, which are minutely analyzed in Technical Document No. 5 [135]. The results demonstrated that the fatigue limits are chiefly affected by tensile strength and hardness. Figure 2 shows typical results that indicate the relationship between the fatigue limits and the tensile strengths. The fatigue limits thus increase as a function of tensile strength. This means that the fatigue limits can be estimated if the tensile strength is known. On the other hand, the effects of microstructures can also be seen in Figure 2, i.e., tempered martensite shows superior fatigue limits to ferrite/pearlite. When estimating the fatigue limits, therefore, the microstructural effects should be taken into account.

**Figure 3.**
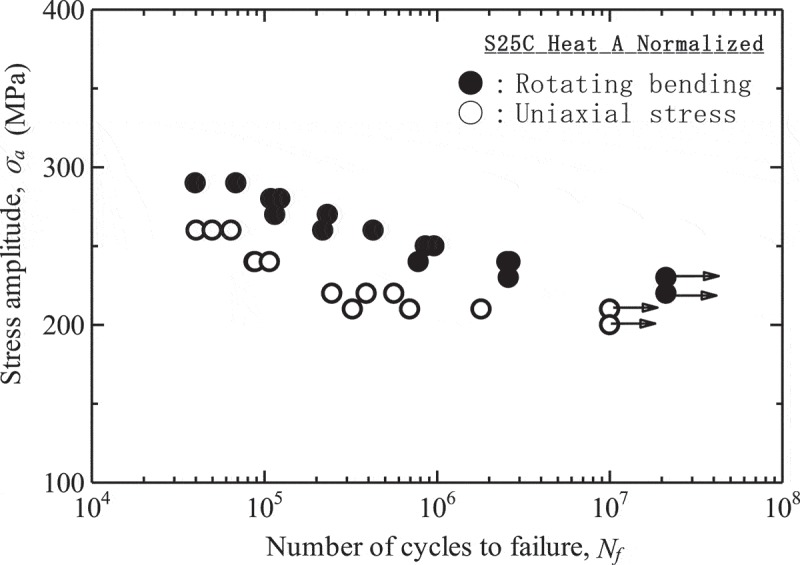
Comparison of high-cycle fatigue test results between rotating bending fatigue tests and uniaxial stress fatigue tests. The plots with arrows indicate runouts.

The high-cycle fatigue properties are also affected by loading modes. Figure 3 shows a comparison of the results between the rotating bending fatigue tests and the uniaxial stress fatigue tests. As seen in Figure 3, rotating bending shows higher fatigue strength than does uniaxial stress. This difference, which is caused by the effects of stress gradients, is more notable in low-strength materials. In other words, stress gradients have only minor effects in high-strength materials.

**Figure 4.**
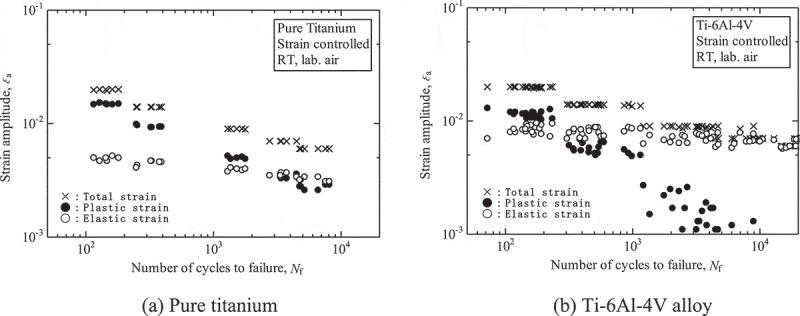
Examples of constant strain amplitude test results.

The low-cycle fatigue properties chiefly reveal fatigue damage caused by cyclic plastic deformation. Figure 4 shows examples of constant strain amplitude test results. The total strain, which is controlled in low-cycle fatigue tests, is divided into plastic and elastic strains. The high strain regions in which the plastic strains exceed the elastic strains are the low-cycle fatigue regions to which the Manson-Coffin law applies. The low-cycle fatigue regions vary with the ductility of the materials, i.e., the region is wide in ductile materials such as pure titanium, while it is narrow in less ductile materials such as Ti-6Al-4V alloy.

**Figure 5.**
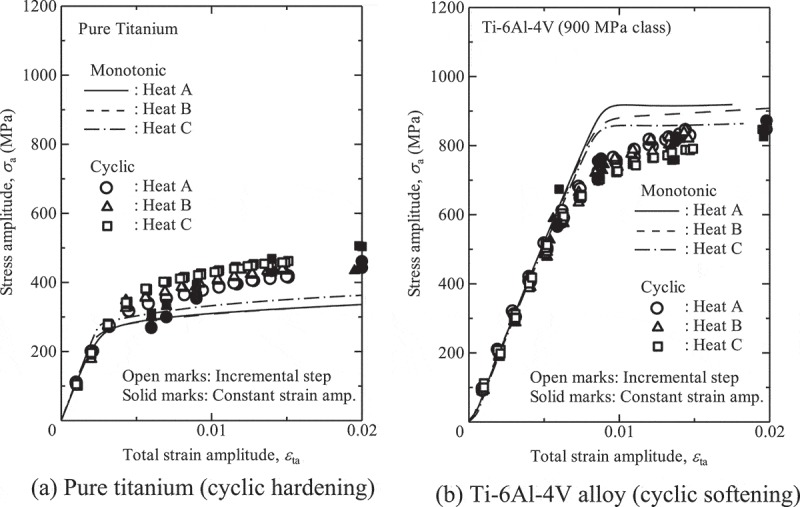
Examples of cyclic stress-strain curves.

Cyclic stress-train curves determined by incremental step tests are another important data set in the low-cycle fatigue tests. Cyclic plastic deformation causes microstructural changes, resulting in the occurrence of cyclic softening or hardening. Figure 5 shows examples of cyclic stress-strain curves. Low-strength, ductile materials such as pure titanium frequently reveal cyclic hardening. On the other hand, high-strength and less ductile materials such as Ti-6Al-4V alloy are frequently subject to cyclic softening.

**Figure 6.**
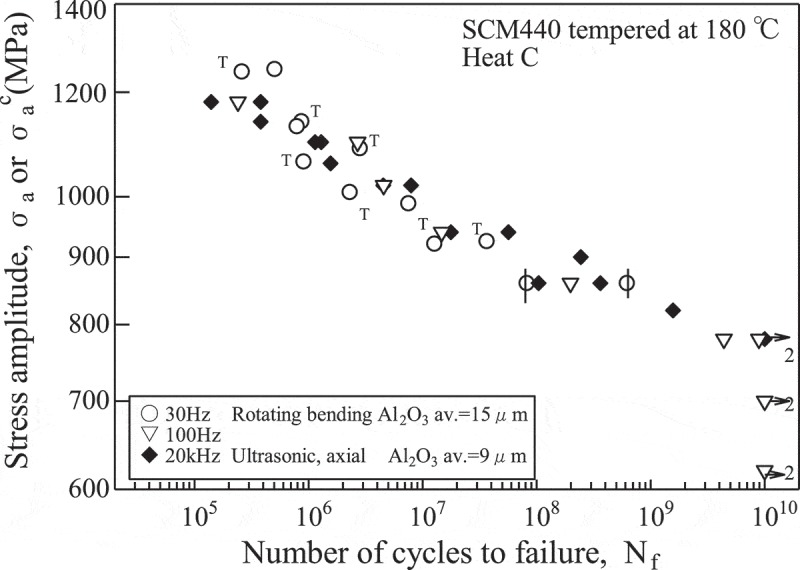
Typical results of gigacycle fatigue tests. The plots with ‘T’ indicate internal fractures originating from TiN inclusions and those with vertical bars ‘|’ indicate surface fractures. Others indicate internal fractures originating from oxide-type inclusions. The number beside the arrows indicate the number of overlapped specimens.

The gigacycle fatigue properties were evaluated for high-strength steels, Ti-6Al-4V alloys and aluminium alloys. Although fatigue cracks normally initiated at surfaces, i.e., surface fractures occurred, the high-strength steels revealed internal fractures when the fatigue limits disappeared. This was also the case for Ti-6Al-4V alloys. Under conditions where internal fractures occurred, ultrasonic fatigue testing showed good agreement with conventional fatigue test results. Figure 6 shows typical results comparing ultrasonic fatigue testing with rotating bending fatigue testing. The rotating bending tests at 100 Hz were conducted for three years to reach 10^10^ cycles. The frequency effects are thus negligible under conditions where internal fractures occur, and ultrasonic fatigue testing can therefore be applied to the gigacycle fatigue tests on high-strength steels and Ti-6Al-4V alloys. With aluminium alloys, although internal fractures were rare, ultrasonic fatigue testing results showed good agreement with those of conventional fatigue testing. These results support the use of ultrasonic fatigue testing and contribute to the establishment of a standard for ultrasonic fatigue testing [134].

**Figure 7.**
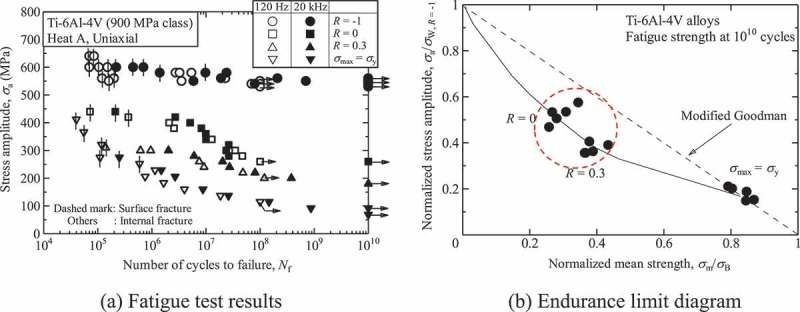
Stress ratio effects on gigacycle fatigue properties of Ti-6Al-4V alloys.

The gigacycle fatigue properties were also evaluated under high stress ratio conditions. The stress ratio effects need careful attention, particularly with Ti-6Al-4V alloys. Figure 7 shows typical results revealing the stress ratio effects. The Ti-6Al-4V alloys are more prone to internal fractures under high stress ratio conditions, resulting in major degradation of the gigacycle fatigue strengths. As a result, the Ti-6Al-4V alloys showed lower fatigue strengths than their modified Goodman lines. In general, modified Goodman lines provide predictions that have a wide safety margin, so these results are unusual. This phenomenon is seen uniquely in gigacycle fatigue, since conventional 10^7^-cycle fatigue strengths show good agreement with the modified Goodman lines [136].

**Figure 8.**
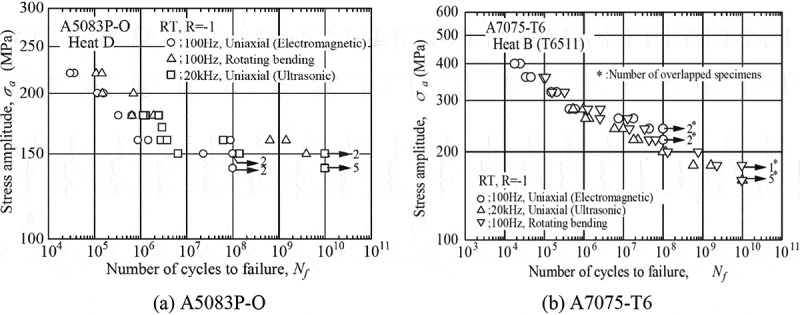
Typical gigacycle fatigue test results for aluminium alloys.

Figure 8 shows typical gigacycle fatigue test results for aluminium alloys. The rotating bending tests at 100 Hz were conducted for three years to reach 10^10^ cycles, as well as the ultrasonic fatigue tests at 20 kHz for a week. Although these alloys have been regarded as materials that show no fatigue limit, the A5083P-O alloy clearly shows them. The fatigue limits are in fact obscure when the fatigue tests are terminated at 10^7^ cycles, but they are confirmed by 10^10^-cycle fatigue tests. On the other hand, no fatigue limits are observed in A7075-T6 alloy. The presence of the fatigue limits thus depends on the type of aluminium alloy, so further gigacycle fatigue tests are necessary for other types of aluminium alloys to clarify the presence or absence of fatigue limits. Accordingly, A6061-T6 alloy was selected as the next material of the gigacycle fatigue tests, and the results will appear in future in the fatigue data sheets.

### 3.2. Fatigue properties at high temperatures

**Figure 9.**
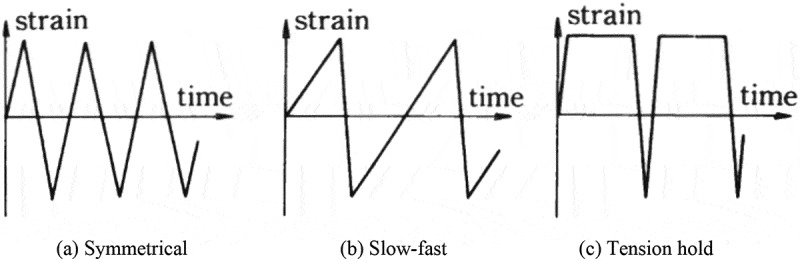
Waveforms employed in constant strain amplitude tests at high temperatures.

**Figure 10.**
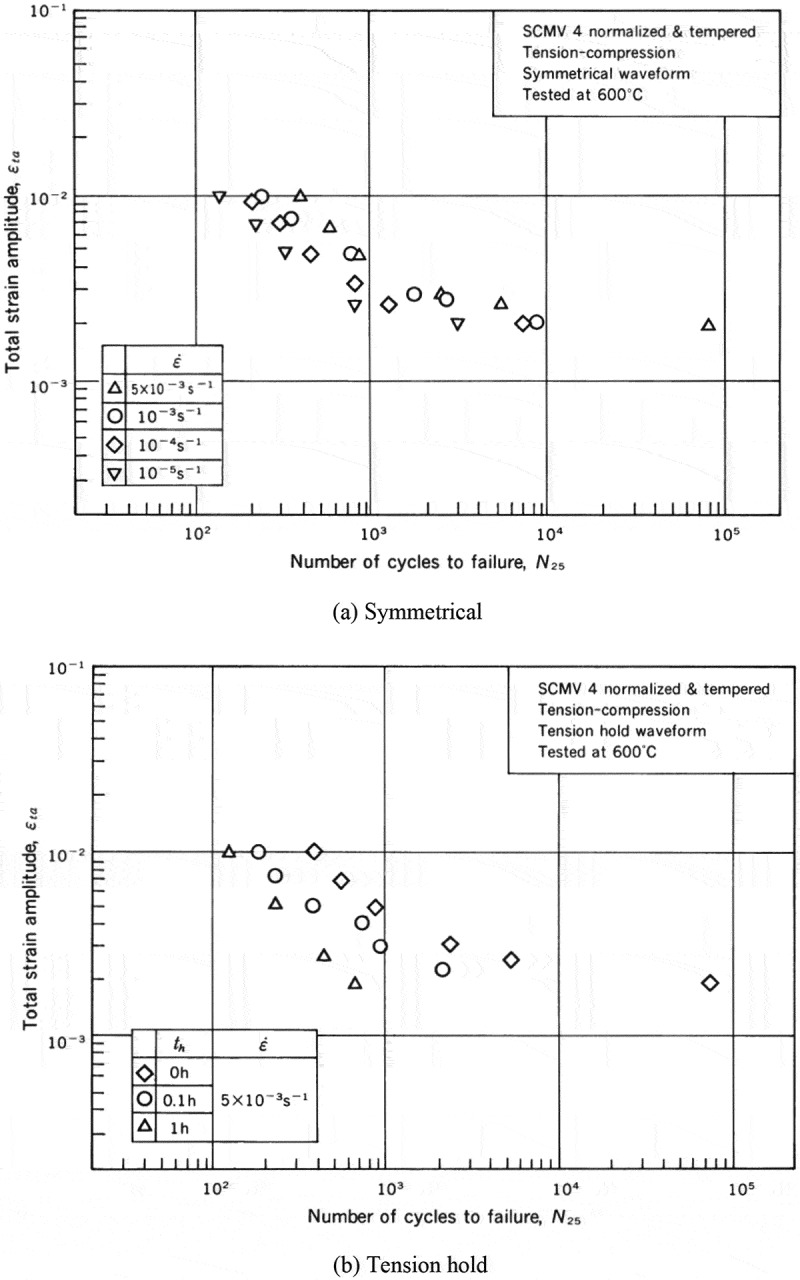
Example of the constant strain amplitude test results at high temperatures. The original images of this figure can be seen in ref [81].

**Figure 11.**
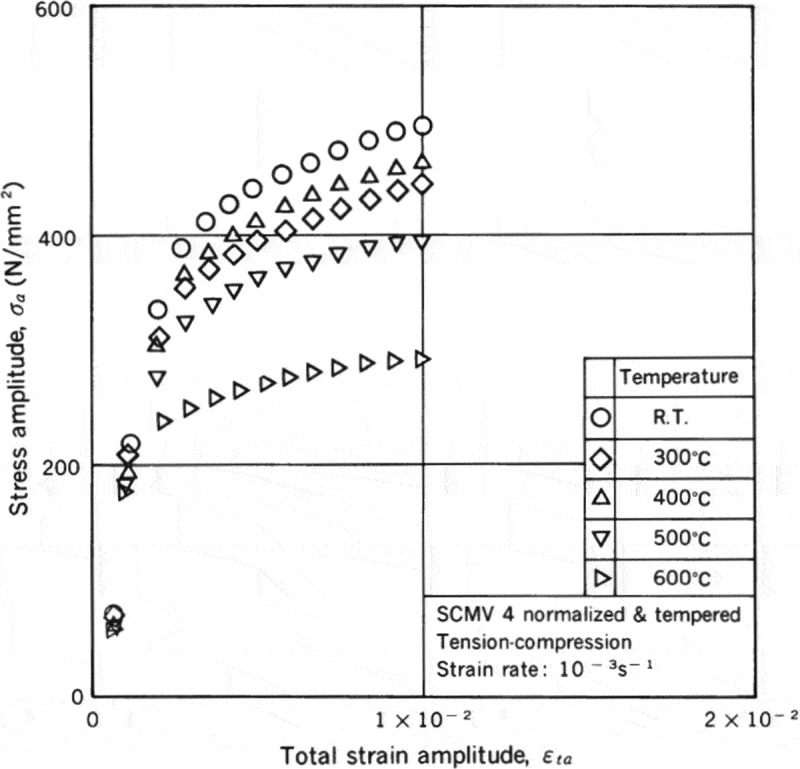
Example of the incremental step test results at high temperatures. The original image of this figure can be seen in ref [81].

The low-cycle fatigue properties at high temperatures were evaluated by employing various strain rates and waveforms. The strain rates range from 10^–3^ to 10^–5^ s^–1^. The waveforms are shown in Figure 9. Figure 10 shows examples of the results. The effects of the strain rates and the waveforms are more visible at high temperatures. These data are minutely analyzed in technical document No. 11 [137]. In addition to these constant strain amplitude tests, incremental step tests were conducted to clarify cyclic stress-strain curves at high temperatures. Figure 11 shows examples of cyclic stress-strain curves. These data are very practical and useful in designing actual high temperature components.

**Figure 12.**
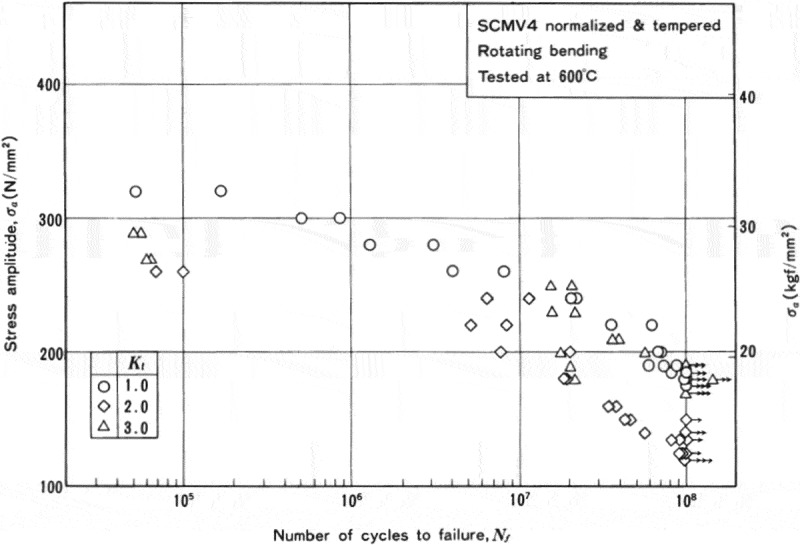
Example of the high-cycle fatigue test results at high temperatures. The original image of this figure can be seen in ref [77].

**Figure 13.**
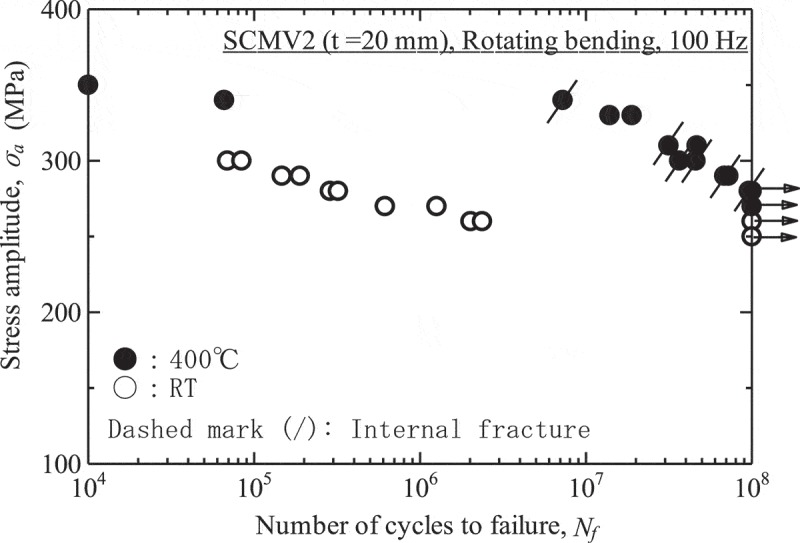
Example of the high-cycle fatigue test results at intermediate temperatures.

The high-cycle fatigue properties at high temperatures illustrate whether or not fatigue limits appear. Figure 12 shows an example of the high-cycle fatigue test results. Even if the materials show fatigue limits at room temperature, they frequently disappear at high temperatures. Internal fractures are also observed in these cases. Figure 13 shows an example of the results at intermediate temperatures. In short-life regions, the fatigue strength at intermediate temperatures shows higher fatigue strength than at room temperature because of cyclic strain hardening, whereas it drops in long-life regions because of internal fracturing.

**Figure 14.**
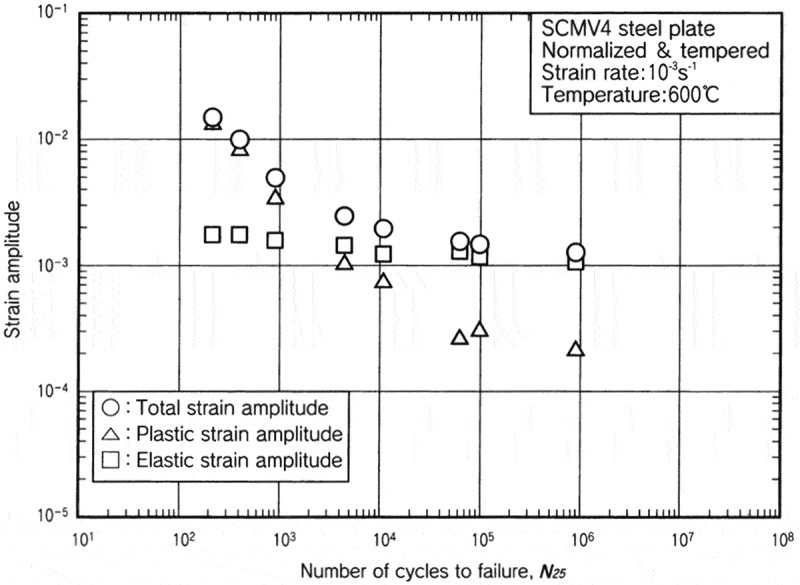
Example of the long-term strain-controlled fatigue test results at high temperatures. The original image of this figure can be seen in ref [94].

Figure 14 shows an example of long-term strain-controlled fatigue test results at high temperatures, which were used to evaluate high-cycle fatigue properties. In general, high-cycle fatigue properties are evaluated using load-controlled tests, but in these tests, strain amplitude is not always constant. Strain- and load-controlled test results can be combined under the assumption that average strain or stress amplitudes can be used; however, this assumption reduces the reliability of the fatigue design curves. The best way to eliminate dependence on this assumption is to carry out long-term strain-controlled fatigue tests. This was why the long-term strain-controlled tests were conducted in spite of the considerable time required.

### 3.3. Fatigue properties of welded joints

**Figure 15.**
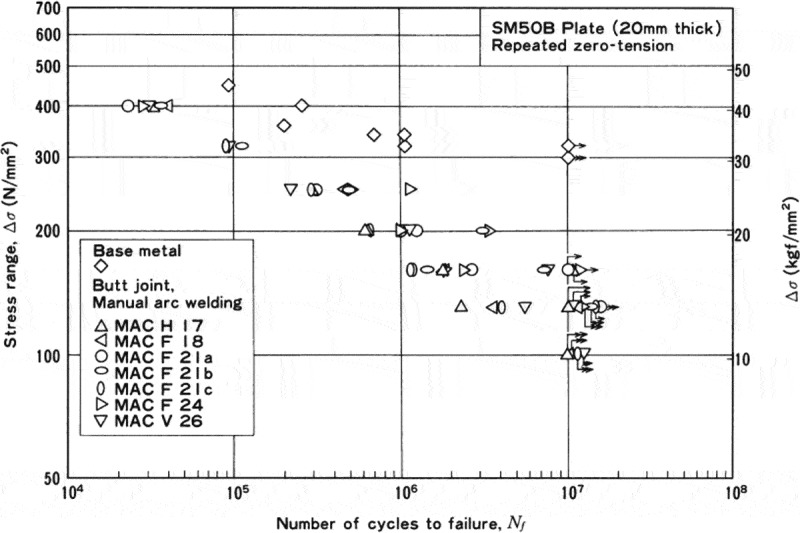
Example of fatigue test results for welded joints to evaluate the effects of welding procedures. The original image of this figure can be seen in ref [106].

The high-cycle fatigue tests for the welded joints were conducted under various conditions to evaluate the effects of specimen size, welding procedures, stress ratios and so on. Figure 15 shows an example of high-cycle fatigue test results employed to evaluate the effects of welding procedures. The mass of data included in the fatigue data sheets is extensive enough to evaluate these effects in detail.

**Figure 16.**
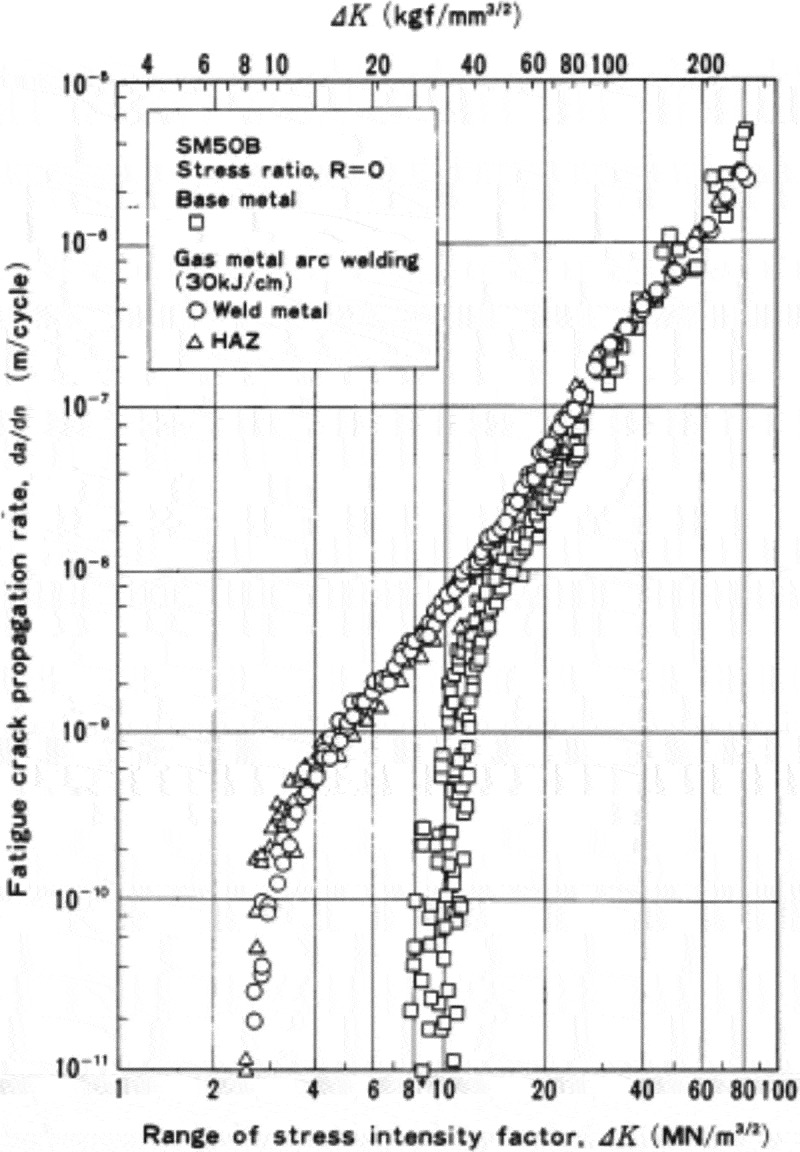
Example of crack propagation test results for welded joints. The original image of this figure can be seen in ref [105].

The fatigue data sheets for welded joints uniquely contain the results of crack propagation tests. Figure 16 shows an example of the crack propagation test results. These were conducted using the large sizes of the centre-cracked tension (CCT) specimens, to identify the crack propagation rates for the base metal, the weld metal and the heat-affected zone.

**Figure 17.**
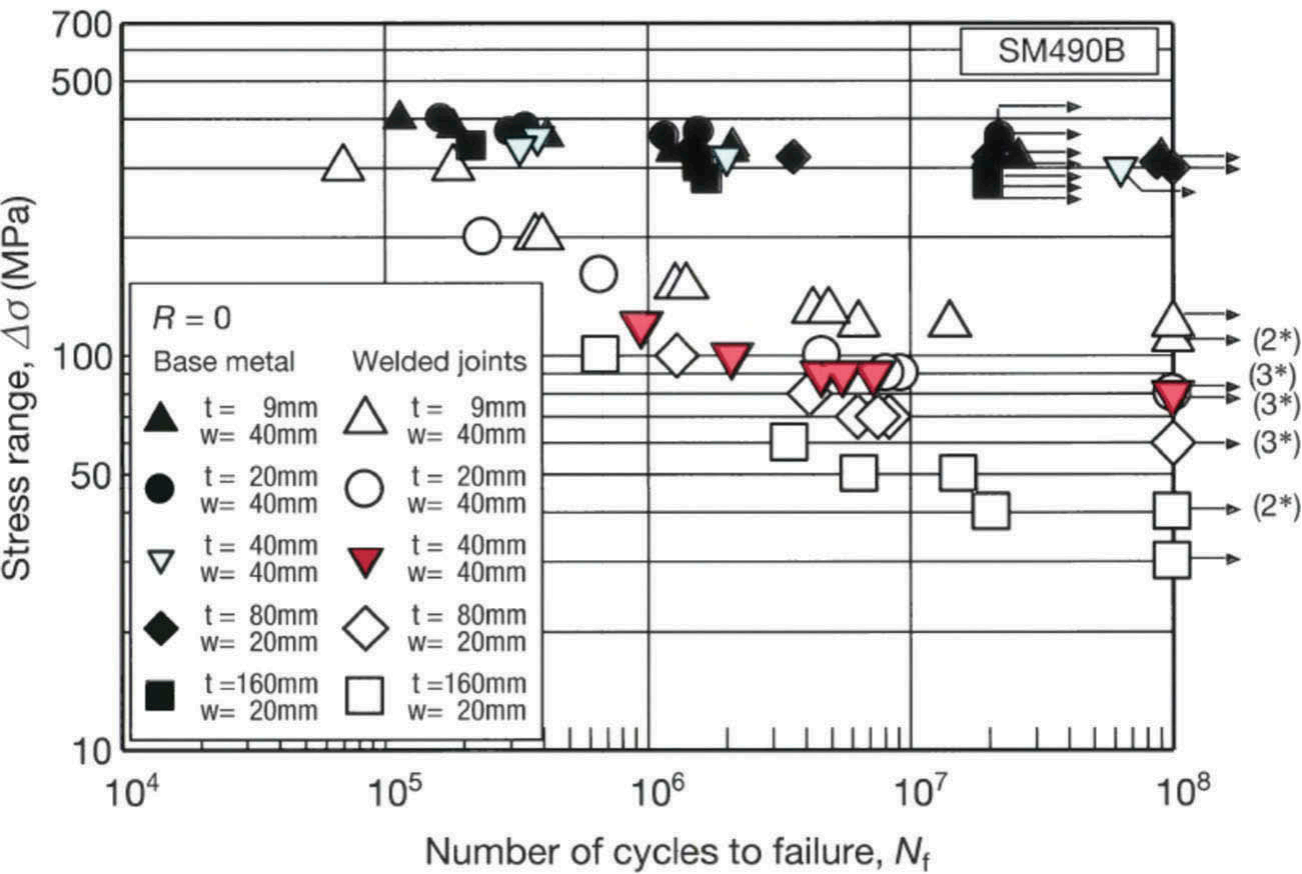
Example of long-term high-cycle fatigue test results for welded joints. The original image of this figure can be seen in ref [126].

Figure 17 shows an example of long-term high-cycle fatigue test results at 10 Hz up to 10^8^ cycles. The specimens for the welded joints are fatigue-fractured at above 10^7^ cycles, so the presence of fatigue limits is not proven, even by 10^8^-cycle fatigue tests. These results mean that the fatigue design curves for the welded joints should not assume the existence of fatigue limits.

## 4. Summary

As of the publication of this review paper, the NIMS fatigue data sheets are fully and freely available on the Internet. The fatigue data sheets are temporally only viewed on ‘MatNavi’ (https://smds.nims.go.jp/fatigue/index_en.html) until the middle of 2020, while they will be fully available on a ‘Materials Data Repository (MDR)’ system managed by NIMS after that. Each data sheet can be reached by using DOI listed in references. They comprise a huge database on the fatigue properties of structural materials. The fatigue data have been constantly added to the database for over 40 years. They cover fundamental fatigue properties at room temperature, fatigue properties at high temperatures, and fatigue properties of welded joints. A recent theme of this project is long-term fatigue, which includes so-called gigacycle fatigue. One major advantage of this database is the very small scattering of the data, because the fatigue tests have been conducted at one institution (NIMS).

The fundamental fatigue properties at room temperature were evaluated for steels, aluminium alloys, titanium alloys and so on. High-cycle, low-cycle and gigacycle fatigue properties were then evaluated. The high-cycle fatigue properties determine the fatigue limits, demonstrating that they are chiefly affected by tensile strength and hardness. The low-cycle fatigue properties reveal not only the fatigue lives under cyclic plastic deformation but also cyclic stress-strain curves. The gigacycle fatigue properties were evaluated by three-year fatigue testing at 100 Hz up to 10^10^ cycles, as well as by ultrasonic fatigue testing at 20 kHz.

Most of the initial data on fatigue properties at high temperatures is for steels. Low and high-cycle fatigue tests were then conducted. The low-cycle fatigue tests at high temperatures were conducted using various strain rates and waveforms, to elucidate their effects. The high-cycle fatigue test results reveal that fatigue limits frequently disappear at high temperatures, in spite of the same materials showing fatigue limits at room temperature. To test long-term fatigue, strain-controlled fatigue tests were conducted up to 10^6^ cycles to evaluate high-cycle fatigue properties under strain-controlled conditions.

The fatigue properties of welded joints were evaluated for steels and aluminium alloys in the form of thick plates. High-cycle fatigue and crack propagation tests were then conducted using large specimens which contained welded parts in as-welded condition. The high-cycle fatigue tests were conducted under various conditions to evaluate the effects of specimen sizes, welding procedures, stress ratios and so on. The crack propagation tests clarified the crack propagation rates for the base metal, the weld metal and the heat-affected zone. For long-term fatigue, high-cycle fatigue tests at 10 Hz were conducted up to 10^8^ cycles, revealing that the presence of fatigue limits is not proven, even in 10^8^-cycle fatigue tests.

The NIMS fatigue data sheets have up to now been used chiefly for industrial purposes. NIMS now intends to promote and extend their use in academia by improving their accessibility. This review paper is the new gateway for accessing the NIMS fatigue data sheets.
